# Impact of Low-Dose X-Ray Radiation Exposure on Lung Cancer Progression

**DOI:** 10.3390/ijms27188272

**Published:** 2026-09-17

**Authors:** Zhijie Chen, Xinxin Guo, Shijia Wang, Peiyu Zhang, Ni Chen, Xuanyu Wang

**Affiliations:** Teaching and Research Section of Nuclear Medicine, School of Basic Medicine, Anhui Medical University, Hefei 230032, China; 2445010109@stu.ahmu.edu.cn (Z.C.); 2445010108@stu.ahmu.edu.cn (X.G.); 2545010111@stu.ahmu.edu.cn (S.W.); hi_gril@126.com (P.Z.)

**Keywords:** low-dose radiation, lung cancer, tumor pulmonary colonization, DNA damage, EMT

## Abstract

Low-dose ionizing radiation is known to alter the biological properties of tumor cells. Although clinical computed tomography delivers low-dose radiation, the long-term radiobiological impacts of repeated low-dose X-ray exposure on the malignant progression of lung adenocarcinoma remain poorly defined and require further preclinical clarification. In this preclinical study, we established long-term passaged A549 lung adenocarcinoma cell models subjected to single and fractionated low-dose X-ray irradiation. In vitro functional phenotypes and molecular alterations were further evaluated using murine subcutaneous xenograft and tail vein pulmonary colonization models to assess tumor growth and lung colonization capacity. Long-term low-dose irradiation significantly promoted lung cancer cell proliferation and migration in vitro and enhanced tumor growth and pulmonary colonization in mice. The results for cells cultured in vitro showed that low-dose radiation induced persistent DNA damage responses, modulated p53 expression, and upregulated the expression of epithelial–mesenchymal transition (EMT)- and glycolysis-related proteins, thereby remodeling the aggressive malignant phenotype of lung adenocarcinoma cells. Prolonged low-dose X-ray exposure drives progressive malignant phenotypic remodeling in A549 cells. Our work provides experimental radiobiological evidence of radiation-promoted tumor progression. Importantly, these preclinical findings are restricted to cell culture and animal models and cannot be directly extrapolated to clinical situations.

## 1. Introduction

Lung adenocarcinoma constitutes the predominant histological subtype of non-small cell lung cancer, and patient prognosis is largely dictated by the invasive and metastatic trajectory of the tumor [1]. In the era of precision medicine, computed tomography is indispensable for diagnosis, surveillance, and particularly lung cancer screening. However, this clinical dependence has resulted in a significant rise in iatrogenic radiation exposure. Epidemiological data indicate a substantial increase in the detection rate of pulmonary nodules, which correlates with the millions of CT examinations performed annually [2,3,4]. Consequently, a growing demographic of patients is accruing cumulative effective doses exceeding 250 mSv over repeated diagnostic intervals [4]. While the individual dose from a single diagnostic CT, typically 2 to 20 mSv, or a low-dose screening CT of approximately 1.5 mSv is relatively modest, the cumulative biological impact of these repetitive low-dose exposures on existing malignancies remains a critical area of investigation.

The radiobiological implications of low-dose radiation are historically contentious. Although international guidelines generally classify exposures below 100 mSv as low-dose radiation, a threshold implying that deterministic effects are absent and stochastic risks are often considered negligible or extrapolated via the linear no-threshold model, emerging evidence challenges the assumption of biological neutrality [5,6]. Epidemiological cohorts, particularly those involving pediatric and adolescent exposures, have demonstrated dose-dependent associations between diagnostic radiation, such as cumulative doses of approximately 50 mGy, and an elevated risk of hematological and solid malignancies [7,8]. Patients with established malignancies who have received prior radiotherapy, chemotherapy, or interventional procedures should receive limited serial low-dose CT surveillance to reduce cumulative low-dose radiation burden [9]. Even clinically relevant low-dose fractions were found to possess significant genotoxic potential capable of inducing DNA double-strand breaks and oxidative stress [10,11].

Despite established evidence linking low-dose radiation to carcinogenesis and tumor initiation, accumulating preclinical data demonstrate that chronic low-dose-rate radiation, which recapitulates repeated serial low-dose computed tomography (LDCT) surveillance, drives the progressive malignant transformation of pulmonary epithelial cells. This oncogenic effect is mediated by sustained genomic instability, epithelial–mesenchymal transition (EMT), and angiogenic signaling cascades governed by the ANGPTL4–SDC4 ligand–receptor axis [12]. Although this work characterized radiation-induced de novo carcinogenesis in normal lung cells, it did not address whether fractionated low-dose CT radiation aggravates the progression of pre-established lung adenocarcinoma, which constitutes the core research gap resolved by our present study. There is a paucity of longitudinal data addressing how repeated exposures influence the progression of established tumors. Specifically, the long-term phenotypic consequences of fractionated low-dose irradiation on lung adenocarcinoma cells are poorly understood. Emerging research suggests that low-dose radiation may modulate the tumor microenvironment and intrinsic cellular signaling, potentially driving phenotypic plasticity [6,13,14,15]. However, most extant experimental studies are limited to short-term observations and fail to capture the persistent evolutionary changes that may arise from chronic diagnostic surveillance [16,17].

To bridge this knowledge gap, the present study evaluates the long-term biological sequelae of clinically relevant low-dose radiation on the malignant phenotype of lung adenocarcinoma cells. We utilized an in vitro model of repeated low-dose X-ray exposure, comparing single versus repeated exposures ranging from 3 to 9 cGy, to investigate the temporal dynamics of DNA damage repair and subsequent phenotypic evolution. Our findings reveal that while low-dose radiation induces transient genotoxicity, its more profound impact lies in driving a long-term adaptive response characterized by enhanced proliferation, the acquisition of epithelial-to-mesenchymal transition features, and increased therapeutic resistance. Furthermore, we corroborate these findings with in vivo assays demonstrating elevated pulmonary colonization capacity. This study provides mechanistic evidence that repeated diagnostic-level radiation is not biologically inert but may actively select for a more aggressive tumor phenotype, underscoring the need for a balanced risk–benefit assessment in radiologic surveillance protocols.

## 2. Results

### 2.1. Low-Dose X-Ray Irradiation Induces DNA Damage

**Figure 1:**
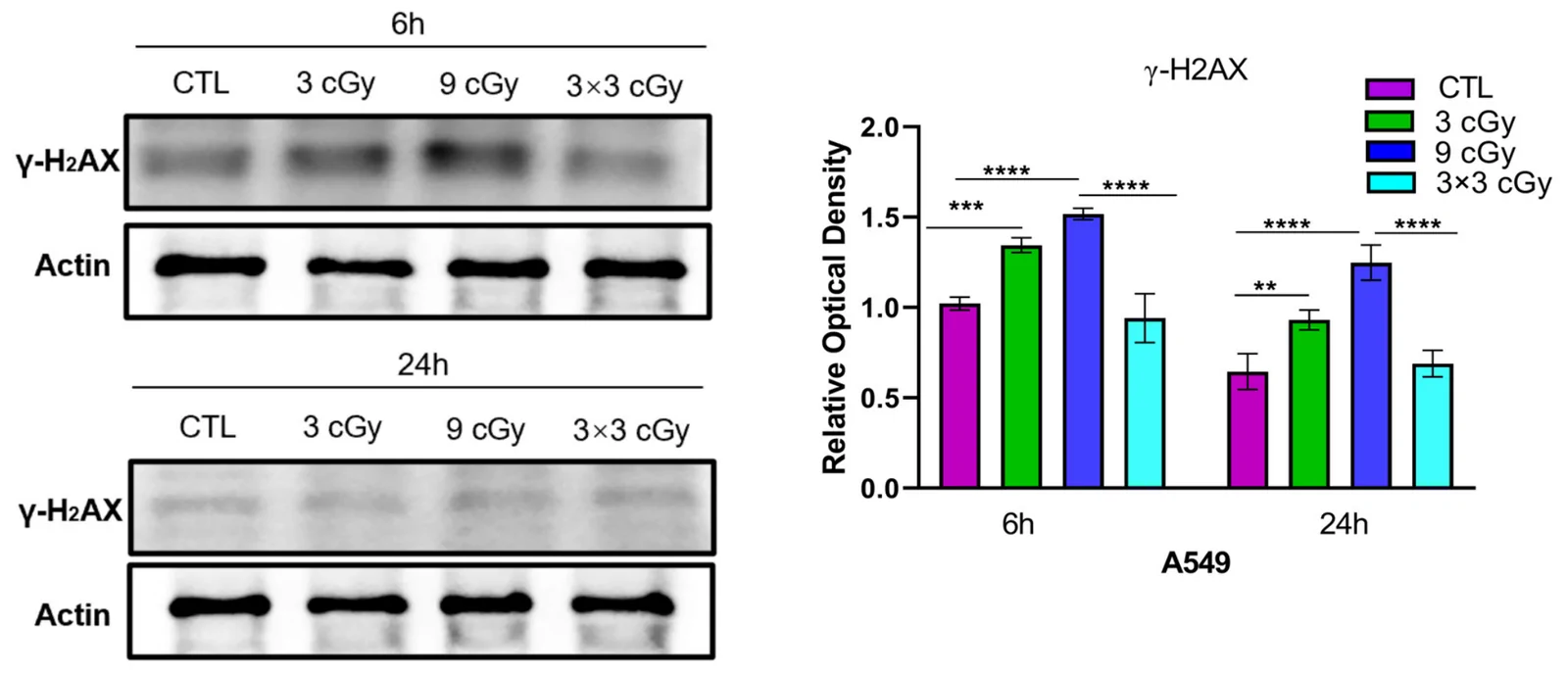
Low-dose X-ray irradiation causes DNA damage and increases γ-H2AX protein phosphorylation. Cells were irradiated in three different dose modalities: one dose of 3 cGy, one dose of 9 cGy, and one dose of 3 cGy per day for three consecutive days (9 cGy in total). Sham-irradiated cells were the control cells. The expression level of the DNA damage biomarker γ-H2AX was detected at 6 h and 24 h after irradiation to evaluate DNA damage responses. *n* = 4. Data are presented as the mean ± SD. ** *p* < 0.01; *** *p* < 0.001; **** *p* < 0.0001.

We examined DNA damage responses in A549 cells by measuring γ-H2AX protein levels at 6 and 24 h after low-dose irradiation. At 6 h post radiation, both the 3 cGy and 9 cGy doses were associated with elevated γ-H2AX phosphorylation compared to controls, with a more pronounced increase observed in the 9 cGy group (Figure 1). By 24 h, γ-H2AX phosphorylation decreased in the single-dose groups but remained higher than that in controls. Notably, the 3 × 3 cGy group showed no significant difference from controls at either 6 or 24 h (Figure 1). These results indicate that single low-dose irradiation induced detectable γ-H2AX signaling, whereas the 3 × 3 cGy fractionated regimen did not induce a significant increase in γ-H2AX at the assessed time points. Therefore, no conclusion regarding persistent DNA damage can be drawn for the 3 × 3 cGy group.

### 2.2. Low-Dose X-Ray Irradiation Alters the Cell Cycle Distribution

**Figure 2:**
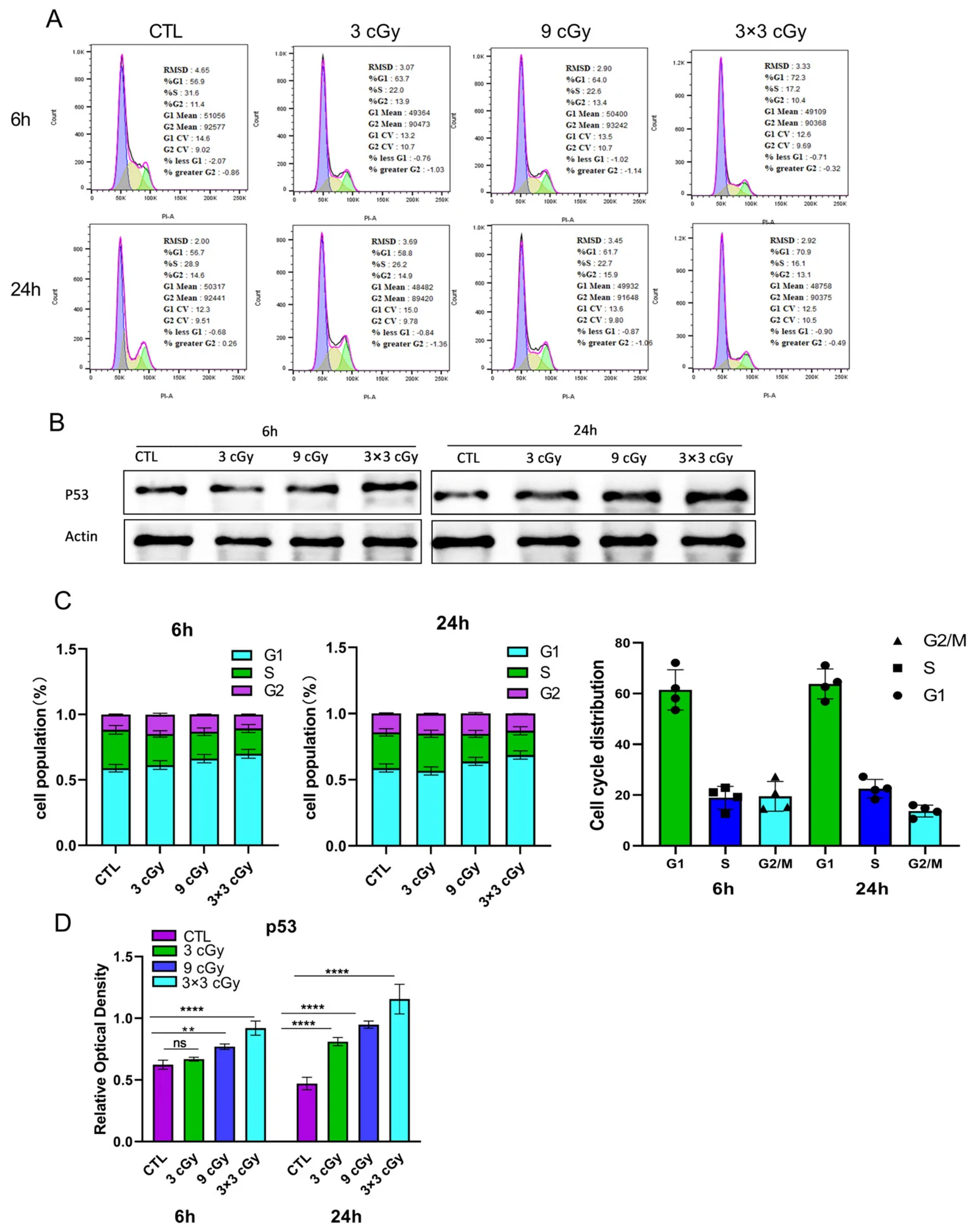
Low-dose X-ray irradiation induces cell cycle arrest and activates DNA damage repair. A549 cells were collected 6 h and 24 h after irradiation with three different dose modalities. (**A**) Flow cytometry histograms of cell cycle distribution and (**C**) statistical analysis of proportion of G2 phase cells; (**B**) expression of p53 protein and (**D**) statistical analysis of relative optical density of p53. *n* = 4. Data are presented as mean ± SD. ** *p* < 0.01; **** *p* < 0.0001; ns, no significant.

Cell cycle analysis (Figure 2A) revealed G2/M arrest in the 3 cGy and 9 cGy groups at 6 h, which was partially restored by 24 h. Notably, the 3 × 3 cGy group displayed persistent G1 arrest at both time points. To explore this further, p53 protein levels were examined (Figure 2B,D). All irradiated groups showed increased p53 expression compared to controls, with the highest levels in the 3 × 3 cGy group, suggesting a link between DNA damage, cell cycle arrest, and p53 activation.

### 2.3. Low-Dose X-Ray Irradiation Stimulates Cell Proliferation

**Figure 3:**
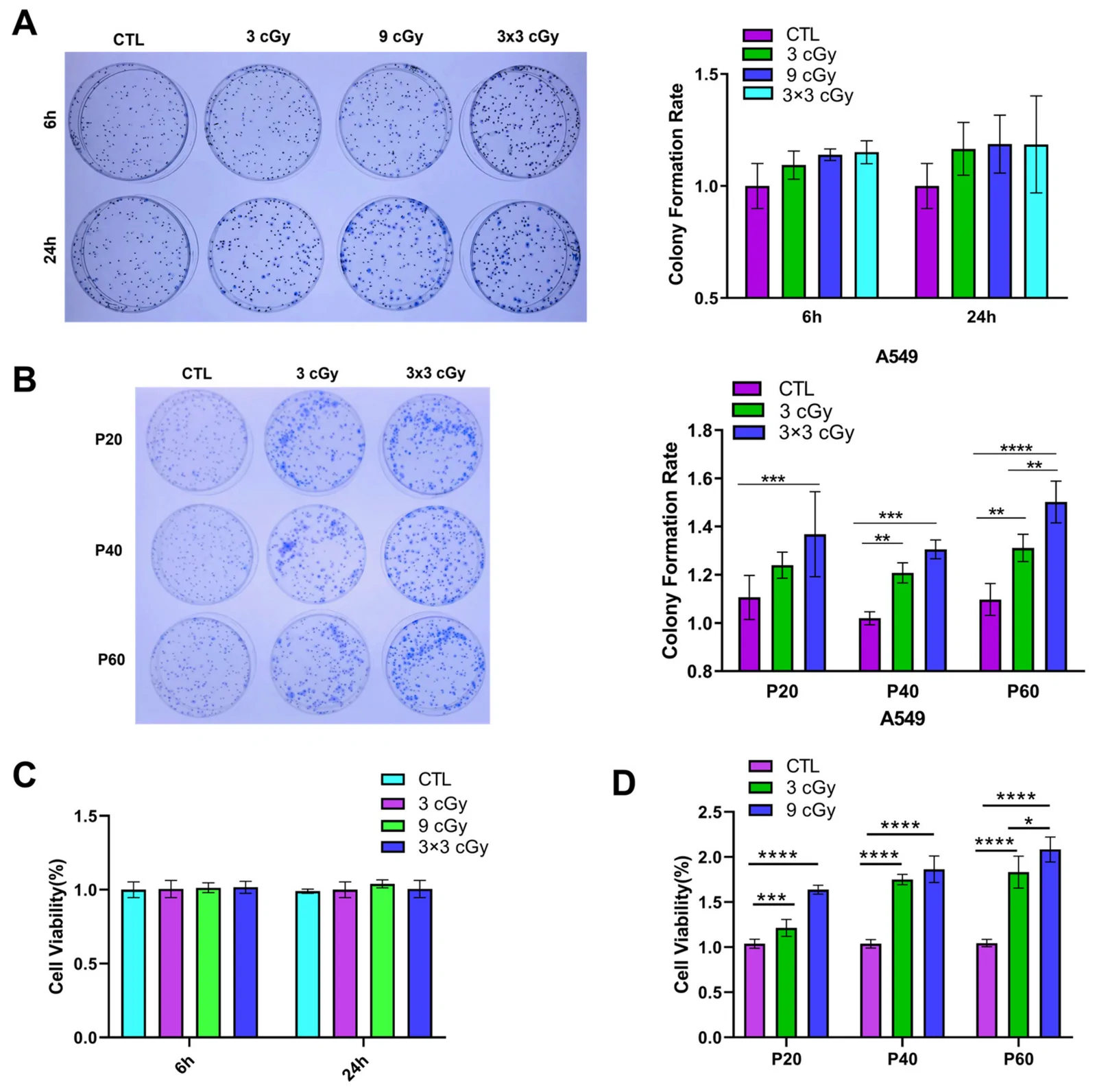
Low-dose X-ray irradiation heightens cell proliferation in CT scan irradiated long-term cultured lung cancer cells. Cells were collected 6 h and 24 h after irradiation. (**A**) shows the results of the colony formation rate, and (**C**) shows cell viability; A549 cells irradiated with three different doses were cultured to 60 generations. The 20th-, 40th- and 60th-generation cells were collected; (**B**) shows the colony formation rate, and (**D**) shows the cell viability of long-term cultured cells. *n* = 3. Data are presented as the mean ± SD. * *p* < 0.05; ** *p* < 0.01; *** *p* < 0.001; **** *p* < 0.0001.

To clarify the effect of different low-dose irradiation doses on the proliferation of A549 cells, the colony formation rate and cell viability were examined. Colony formation and cell viability assays (Figure 3) showed no significant changes 6 or 24 h post low-dose irradiation (3 cGy, 9 cGy, 3 × 3 cGy) compared to controls. However, in long-term cultures, both irradiated groups exhibited increased proliferation and viability with passage number, with the 3 × 3 cGy group showing a stronger effect (Figure 3B,D). These findings suggest that low-dose X-ray irradiation may promote A549 cell proliferation over time.

### 2.4. Increased Cell Viability in Long-Term Cultured Cells

**Figure 4:**
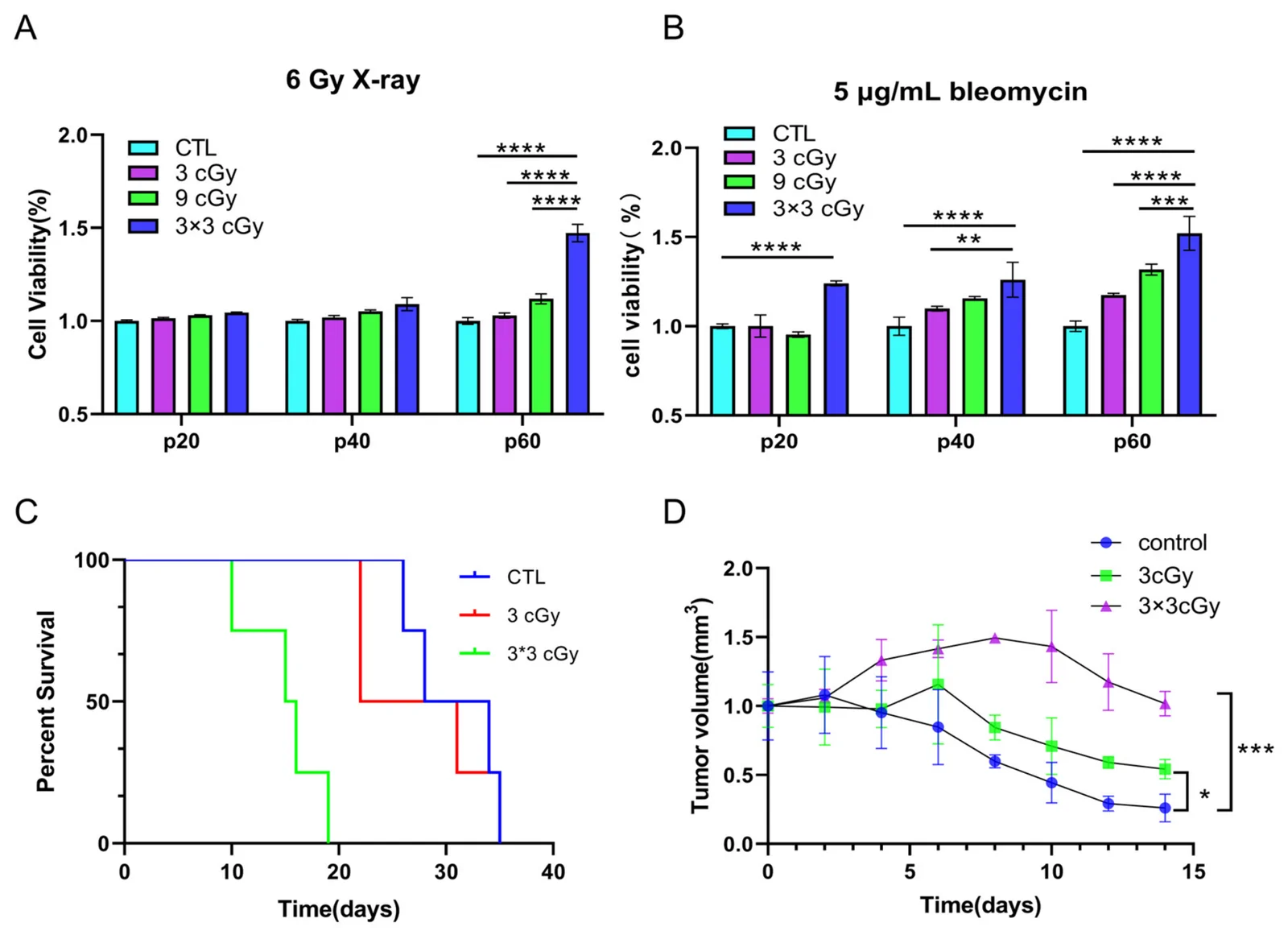
The development of viability in long-term cultured cells after low-dose X-ray irradiation. (**A**) The cell viability of the 40th and 60th generations of cells exposed to 6 Gy X-ray irradiation in the 3 cGy group and the 3 × 3 cGy group. (**B**) The cell viability of the 40th and 60th generation cells treated with 5 μg/mL bleomycin for 1 h in the 3 cGy group and 3 × 3 cGy group. (**C**) Tumor formation in nude mice that were subcutaneously injected with the 40th-generation cells of the respective cultured irradiated group. After the tumor grew out, the tumor was irradiated with 6Gy X-rays. The survival curve and tumor volume (**D**) of nude mice in each group were described. *n* = 3. Data are presented as the mean ± SD. * *p* < 0.05; ** *p* < 0.01; *** *p* < 0.001; **** *p* < 0.0001.

To assess cell viability, cells at passages 20, 40, and 60 were treated with bleomycin or 6 Gy X-ray irradiation, followed by CCK-8 assays (Figure 4A,B). The results showed that cell viability in the 3 × 3 cGy group increased with passage number, suggesting that long-term culture after low-dose irradiation may enhance the malignant potential of tumor cells.

Furthermore, in vivo validation in nude mice (using passage 40 cells) confirmed that low-dose irradiation exhibited enhanced colonization capacity in pulmonary cells. Compared with non-irradiated controls, tumor growth after 6 Gy re-irradiation was significantly suppressed in all pre-irradiated groups. However, among these groups, the 3 × 3 cGy schedule showed the weakest response to re-irradiation, as evidenced by persistently higher tumor volume trajectories, and conferred the strongest tumor aggressiveness, which ultimately correlated with the shortest survival (Figure 4C,D). These findings indicate that long-term culture following low-dose X-ray irradiation significantly elevates the cell viability of tumor cells.

### 2.5. Low-Dose X-Ray Irradiation Increased Metastatic Potential of A549 Cells

**Figure 5:**
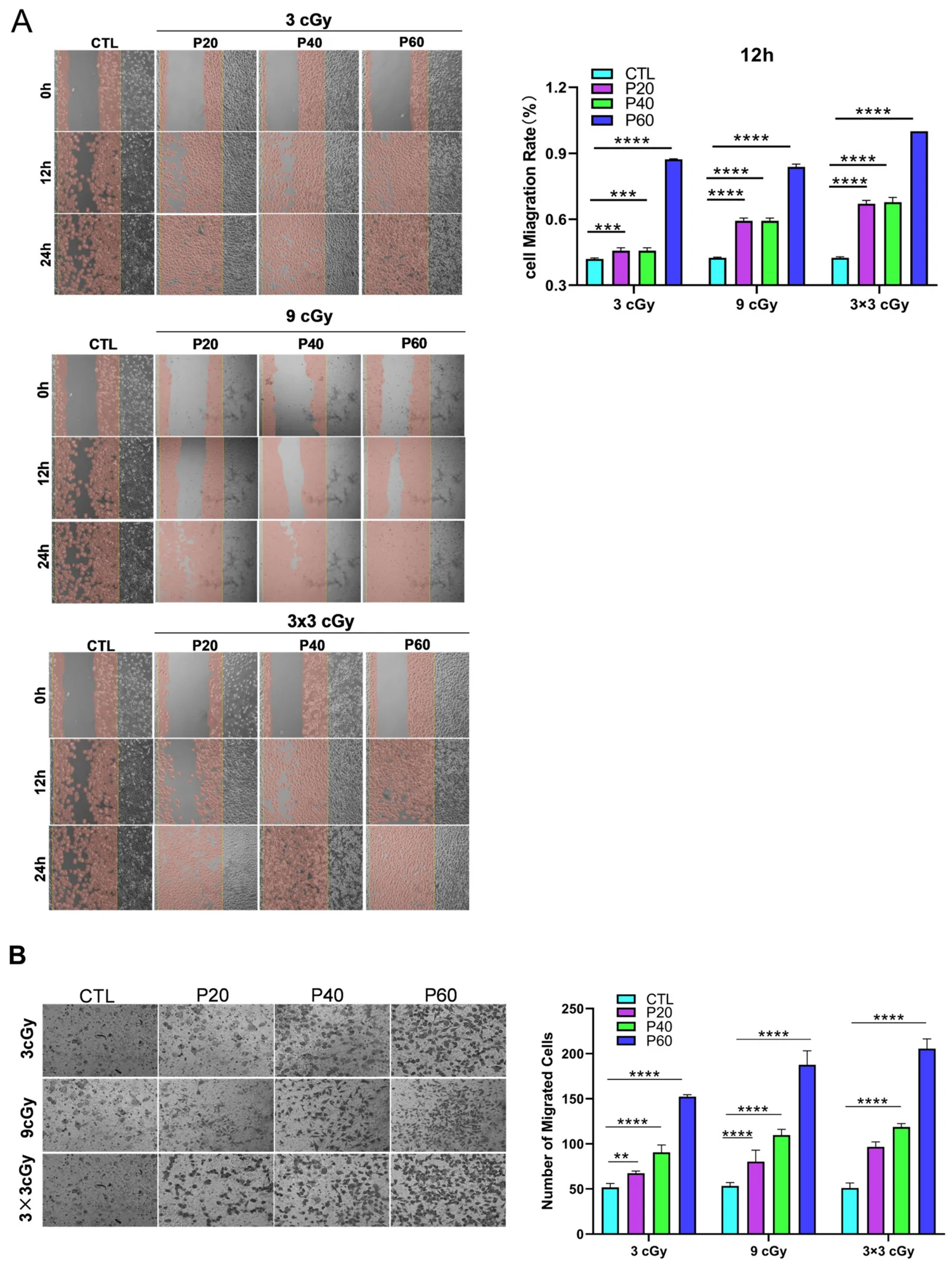
Low-dose X-ray irradiation promotes the metastatic potential of lung adenocarcinoma cells. (**A**) shows the scratch test results of long-term cultured cells in the 3 cGy group and the 3 × 3 cGy group. (**B**) shows the transwell experimental results of long-term cultured cells in the 3 cGy group and the 3 × 3 cGy group. *n* = 3. (C) Data are presented as the mean ± SD. ** *p* < 0.01; *** *p* < 0.001; **** *p* < 0.0001.

Long-term culture following low-dose irradiation enhanced the migratory and invasive abilities of A549 cells (Figure 5). Scratch assays revealed faster wound closure with increasing passage number in both irradiated groups (3 cGy, 9cGy and 3 × 3 cGy). Transwell assays confirmed a passage-dependent increase in transmigration, with the 3 × 3 cGy group showing a more significant effect (Figure 5B). These findings suggest that low-dose irradiation promotes a migratory phenotype in A549 cells.

**Figure 6:**
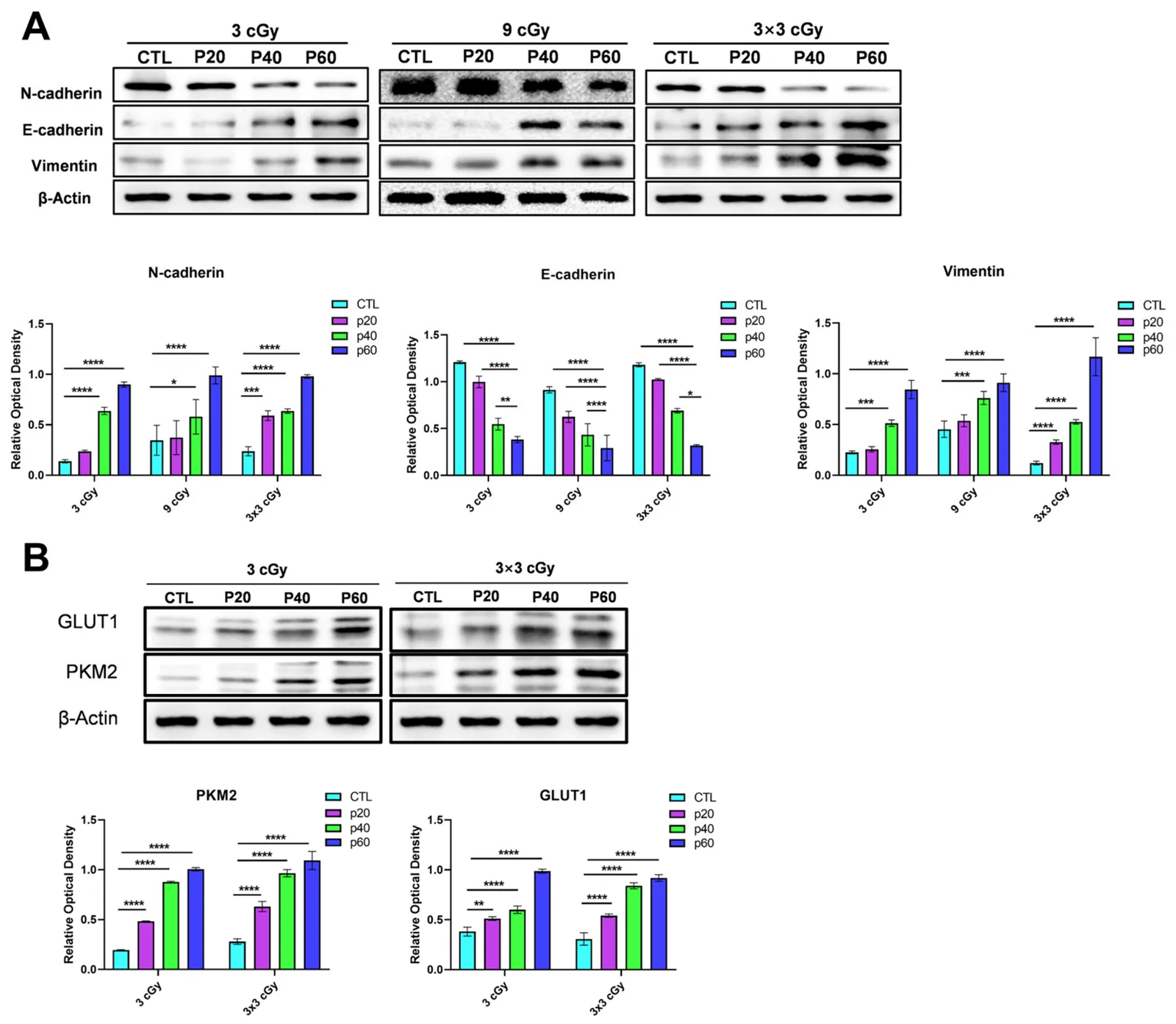
Low-dose X-ray irradiation induces epithelial–mesenchymal transition and increases metabolic protein expression in long-term cultured cells. (**A**) shows the expression levels of E-cadherin, N-cadherin and vimentin in the long-term cultured cells of the 3 cGy, 9 cGy and 3 × 3 cGy groups. (**B**) shows the expression level of glucose metabolism-related proteins glucose transporter type 1 (GLUT1) and the pyruvate kinase M2 (PKM2) protein in the3 cGy group and the 3 × 3 cGy group. *n* = 3. Data are presented as the mean ± SD. * *p* < 0.05; ** *p* < 0.01; *** *p* < 0.001; **** *p* < 0.0001.

Western blot analysis detected altered expression levels of epithelial–mesenchymal transition (EMT) markers following low-dose irradiation (Figure 6A). Compared with the control group, irradiated A549 cells exhibited decreased expression levels of the epithelial marker E-cadherin and increased expression levels of the mesenchymal markers N-cadherin and vimentin in a passage-dependent manner, indicating an EMT-associated phenotypic alteration. Additionally, low-dose irradiation promoted the expression of glucose transporter molecules and glycolytic enzyme PKM2 (Figure 6B), suggesting enhanced metabolic reprogramming to support the observed aggressive phenotype.

### 2.6. Colonization Capacity in Pulmonary Cells After Low-Dose X-Ray Irradiation

**Figure 7:**
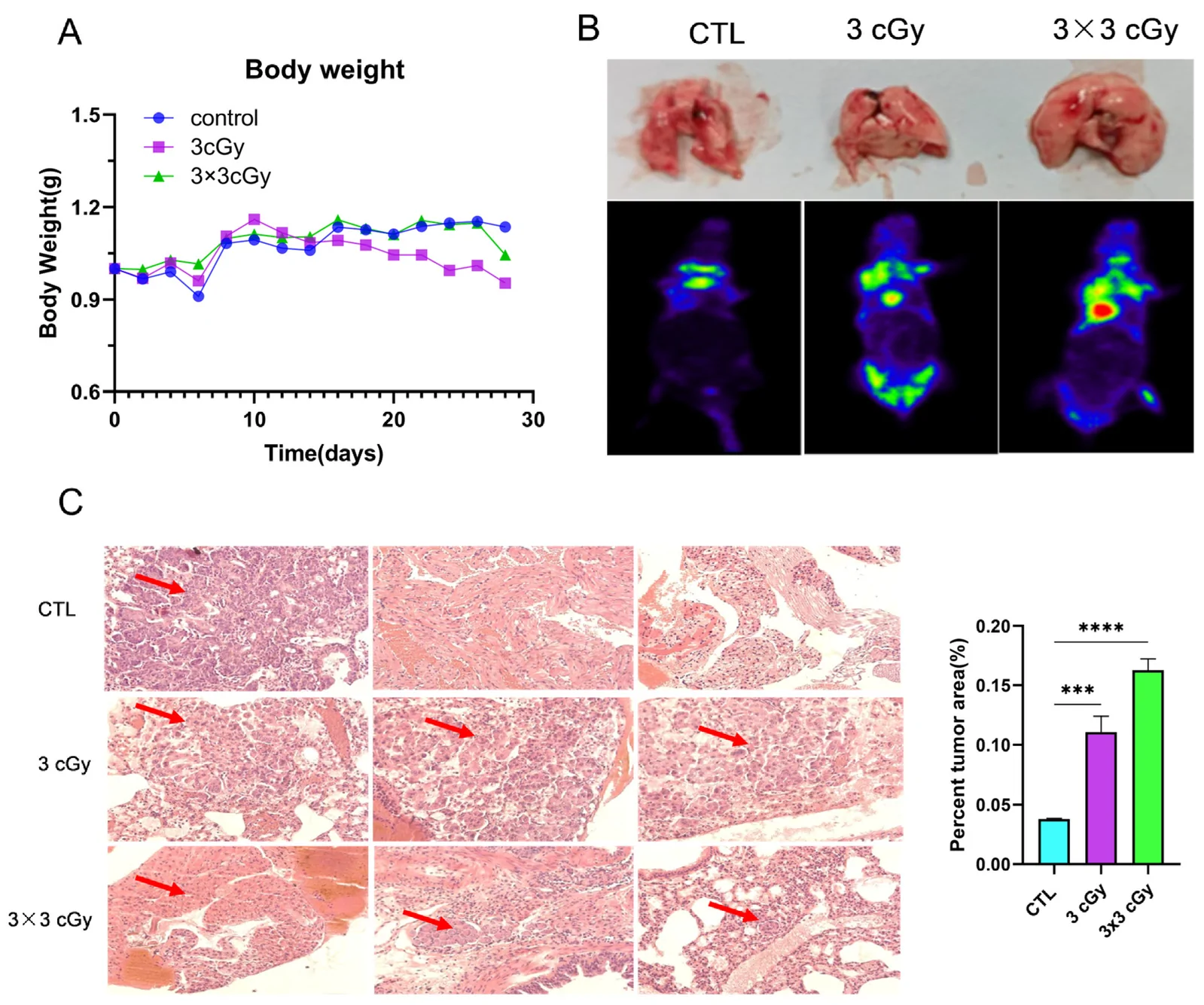
The increased formation of metastatic micronodules in the lungs after low-dose X-ray irradiation. (**A**) shows nude mouse weight changes post the tail vein injection of the 40th generation of cells, and the pathological analysis of a lung tissue section. *n* = 4 mice per group. (**B**) shows the clinical PET imaging of mice. (**C**) H&E lung sections from tumor-bearing mice. *n* = 3. Data are presented as the mean ± SD. *** *p* < 0.001; **** *p* < 0.0001.

Next, we selected the 40th passage of cultured cells for tail vein injection to construct an in vivo tumor lung colonization model in mice. We detected body weight loss in mice about 30 days post tail vein injection, and clinical PET/CT imaging revealed a weak ^18^F-FDG signal in mice bearing sham-irradiated cells compared with that from both irradiated groups. However, the metastatic foci cannot be clearly identified (Figure 7B). Actually, the technique is based on the assessment of tumor glycolysis, which is closely related to GLUT-1 and the hexokinase II (HK-II) enzyme [18]. Pathological analysis clearly showed that cancer cell clusters were scattered in lung tissue, showing the increased colonization ability of both low-dose irradiation groups compared with the control group (Figure 7C). These in vivo results showed that lung adenocarcinoma cells cultured for a long time may enhance tumor malignant transformation after low-dose irradiation. Compared with PET/CT imaging, histological examination served as a more reliable reflection of the true pulmonary tumor burden.

## 3. Discussion

The growing use of CT scans, a major source of diagnostic radiation exposure (14%), raises concerns about potential health risks, particularly for lung cancer patients. This study investigates the effects of low-dose radiation on lung cell growth using the A549 lung adenocarcinoma cell line. It is important to note that the International Atomic Energy Agency defines “low-dose” for exposed populations as radiation below 100 mGy [19]. This study was established with one group receiving a single 3 cGy irradiation. Therefore, the cells in the second group received a single irradiation dose of 9 cGy. Given that the majority of radiation-induced DNA damage is thought to be repaired within 24 h post irradiation [20], we designed a fractionated irradiation regimen consisting of three consecutive daily doses of 3 cGy at 24 h intervals; however, repair was not complete, as residual γ-H2AX signals remained detectable at 24 h. The third group was irradiated for three consecutive days, receiving 3 cGy irradiation every 24 h.

The phosphorylation of γ-H2AX is the most sensitive marker of DSB production. DNA damage caused by low-dose radiation can be quantified through the formation and disappearance of γ-H2AX foci [21,22]. The formation of intracellular γ-H2Ax foci exhibits a dose-linear response in the dose range of a few mGy to 1000 mGy [23]. In determining the fate of cells, the repair of DSBs is even more worthy of attention than the induction of DSBs. It has been reported that only high doses (0.25–1.0 Gy) can cause cell damage, and cells can repair themselves within 24 h of damage, while extremely low (0.01–0.04 Gy) doses of irradiation did not cause any damage to cells [21]. Surprisingly, the repair time of cells after low-dose X-ray irradiation was found to be much longer than that after receiving high-dose radiation during radiotherapy [24,25]. The in vitro irradiation of lymphocytes showed that after 5 h of high-dose (500 mGy) irradiation, 90% of γ-H2AX focal aggregation disappeared with repair, while for low-dose (5 mGy) irradiation, γ-H2AX focal aggregation only decreased by 50% after 5 h of irradiation [21]. In our experiments, γ-H2AX phosphorylation levels at 6 h post irradiation were significantly elevated in both single-dose groups (3 cGy and 9 cGy), with a more pronounced increase in the 9 cGy group. At 24 h, γ-H2AX phosphorylation declined but remained above control levels in both groups. Notably, the 3 × 3 cGy fractionated group showed no significant difference from controls at either time point. However, p53 protein expression was significantly elevated in all irradiated groups, with the highest level observed in the 3 × 3 cGy group, despite the absence of detectable γ-H2AX elevation. γ-H2AX not only participates in the recognition and repair of DSBs but also interacts with p53 to activate cell cycle checkpoints, thereby gaining time for DNA repair and ensuring the maintenance of genetic information [26]. It is tempting to speculate that different irradiation regimens may trigger distinct DNA damage repair pathways. However, our flow cytometry-based cell cycle data can only demonstrate phenotypic consistency with this hypothesis and cannot provide direct mechanistic evidence, as we did not examine key signaling molecules such as ATM/ATR, CHK1/2, or p21. It should also be noted that γ-H2AX protein levels measured by Western blotting reflect the activation of DNA damage response signaling rather than providing a quantitative measure of DSB number or repair kinetics. Complementary approaches, such as immunofluorescence-based γ-H2AX focal counting or neutral comet assays, are required to confirm the quantitative aspects of DNA damage and repair in future studies.

Although DNA DSBs can be repaired, complete error-free repair cannot be guaranteed even at low doses [27]. Notably, when cells repair DSB damage, mutations sometimes occur. When these mutant cells divide and proliferate, this damage will also expand. So, the deterioration of tumor cells is mainly related to their rapid proliferation, and EMT is closely related to the metastatic potential of cells [28]. In our long-term culture of cells treated with low-dose irradiation, the malignant phenotype of A549 cells increased compared with the control group, as evidenced by heightened anaplasia and migration. Concurrently, the expression of EMT-related proteins increased with the number of passages, and the effect of the 3 × 3 cGy regimen was more obvious than that of 3 cGy. Notably, although the 3 × 3 cGy group did not show significant DNA damage after fractionated irradiation, their proliferation and pulmonary colonization potential were indeed enhanced in long-term culture.

In this paper, the hematoxylin and eosin (H&E) staining of lung tissue showed obvious pulmonary colonization foci, while PET-CT imaging 4 weeks or more after injection did not show visible tumors or pulmonary colonization. Several technical limitations of the ^18^F-FDG PET/CT protocol used in this study should be considered. First, the imaging time point (≥4 weeks after cell injection) may have missed the optimal window for detecting early colonization foci. Second, PET/CT imaging was performed on a clinical human-use scanner rather than a dedicated small-animal PET system; due to the small size of mouse lesions, this clinical device could not provide reliable SUV quantification, and its spatial resolution (~1–2 mm) is likely insufficient to detect early colonization foci below that threshold. Third, the glycolytic activity of pulmonary colonization lesions may differ from that of primary tumors due to microenvironmental factors, leading to reduced FDG avidity. Therefore, the negative PET findings should not be interpreted as evidence of early colonization focal quiescence; rather, they underscore that histology remains the gold standard for detecting low-burden pulmonary colonization in this model. Actually, the technique is based on the assessment of tumor glycolysis, which is closely related to GLUT-1 and the hexokinase II (HK-II) enzyme [18]. Despite these limitations, protein analysis revealed the increased expression of GLUT1 and PKM2, markers of enhanced glucose metabolism, with increasing culture passages, suggesting a link to cell malignancy. H&E staining confirmed tumor formation in lung tissues. The complex relationship between glucose metabolism and cancer aggressiveness necessitates further investigation into how low-dose irradiation impacts these pathways and their potential role in pulmonary colonization. Future research will focus on cellular response to DNA damage, DSB repair mechanisms, and how low-dose radiation ultimately triggers pulmonary colonization capacity through genetic and epigenetic modifications.

It is important to emphasize that the present study is primarily descriptive in nature, and the observed alterations in γ-H2AX, p53, EMT markers, and metabolic proteins should be interpreted as correlates of, rather than established causal drivers of, the aggressive phenotype. While our data consistently demonstrate associations between these molecular changes and enhanced proliferation, migration, and in vivo lung colonization under the specific experimental conditions tested, we cannot rule out the possibility that other untargeted pathways may also contribute to the phenotypic effects. Therefore, we refrain from drawing mechanistic conclusions regarding the direct regulatory roles of these markers. Instead, our findings provide a foundation for hypothesis generation and underscore the need for future functional studies, including targeted pathway inhibition or rescue experiments, to formally establish causality.

Several limitations of the present study should also be acknowledged. First, all in vitro functional experiments were performed exclusively using the A549 lung adenocarcinoma cell line. Owing to the marked inter-tumor heterogeneity of clinical lung adenocarcinoma, the cellular phenotypes observed herein may not be generalizable to other lung cancer cell lines or patient-derived primary tumor cells. Second, X-ray irradiation was administered using a laboratory irradiator rather than clinical CT scanners. The absorbed doses adopted in our study cannot be directly converted to the effective doses of routine clinical CT scans, which limits the direct extrapolation of our findings to clinical practice. Third, cells were maintained for up to 60 passages. Prolonged in vitro culture can induce clonal selection, genomic instability, metabolic adaptation, and phenotypic drift, all of which may independently affect proliferation, migration, and EMT. Because passage-matched sham controls were not available for all passage points, the contribution of passage-dependent effects cannot be fully distinguished from radiation-induced effects. Fourth, a single 9 cGy group was not included in all long-term and animal experiments; therefore, no valid comparison between single 9 cGy and 3 × 3 cGy can be made for those datasets, and the contribution of cumulative dose versus fractionation cannot be distinguished. Consequently, no conclusion regarding fractionation-specific effects can be drawn from these datasets. Fifth, the tail vein injection model mainly assesses pulmonary colonization rather than the complete metastatic cascade. Sixth, no a priori power calculation was performed; sample sizes were based on previous experience and resource availability. Therefore, the present findings should be regarded as exploratory and require validation in larger, adequately powered studies. Finally, the biological relevance of radiation-induced phenotypic changes requires further validation in additional cell lines and in vivo models, and the underlying molecular networks remain to be elucidated.

Notwithstanding these limitations, our work offers meaningful preclinical evidence revealing the tumor-promoting properties of low-dose radiation. It is important to place these findings in clinical context. Large randomized controlled trials have demonstrated that low-dose computed tomography (LDCT) screening significantly reduces lung cancer mortality in appropriately selected high-risk populations. Therefore, the present preclinical findings should not be interpreted as evidence against the established clinical use of LDCT screening but rather as a prompt for further research into the biological effects of repeated low-dose radiation exposure and for optimizing screening strategies while preserving its proven mortality benefit. With these caveats, our study highlights a potential long-term risk of low-dose X-ray exposure that warrants closer attention in radiobiology and clinical practice. In future research, we will conduct further validation with multiple cellular models and clinical tissue specimens to strengthen the translational relevance of our findings.

## 4. Materials and Methods

### 4.1. Cell Culture and X-Ray Irradiation

The human non-small cell lung cancer cell line A549 (SCSP-503) was obtained from the Cell Bank of the Chinese Academy of Sciences (Shanghai, China). The cell line was authenticated by short tandem repeat (STR) profiling and confirmed to be free of mycoplasma contamination. Cells were cultured in Dulbecco’s Modified Eagle Medium supplemented (DMEM, 61965059, Gibco, Paisley, UK) with a 1% penicillin–streptomycin mixture (15140-122, Gibco) and 10% fetal bovine serum (FBS, 10270-106, Gibco), followed by incubation at 37 degrees Celsius in an atmosphere maintained at 5% CO_2_. Irradiation was performed using a biological SHARP100 X-ray irradiator (Raycision Medical Technology Co., Ltd. Hefei, Anhui, China) equipped with a tungsten-target X-ray tube (maximum 225 kV, water-cooled, 0.8 mm beryllium window). The dose rate was 0.01 Gy/min. The source-to-sample distance (SSD) was set to 40 cm at an operating tube voltage of 225 kV, with a 20 cm × 20 cm irradiation field. Fitted with a flattening filter based on clinical radiotherapy techniques, the system delivered an irradiation homogeneity of ≥98%. For cellular irradiation, cell culture dishes were positioned on the sample stage, and cells were irradiated perpendicularly by the X-ray beam. Cells were divided into control and irradiated groups (3 cGy, 9 cGy, or 3 cGy × 3 days) after reaching logarithmic growth. Following irradiation and subculture, cells were counted (350,000) at 80–90% confluence and cultured to generation 60. Generations 20, 40, and 60 were chosen for testing.

### 4.2. Cell Colony Formation Assay

Log-phase cells were trypsinized (C0201, Beyotime, Shanghai, China), washed with phosphate-buffered saline and collected. After gradient dilution, 500 cells were seeded in culture plates, and growth was stopped after continuous culture for 8–10 days. After that, the cells were fixed using formalin. Subsequently, cells were stained with crystal violet stain (C8470, Solarbio, Beijing, China) and washed to remove unbound stains. The number of colonies was counted using a colony counting plate (CO010101, Shanghai, China). A cell cluster of >50 cells was counted as a clone. GraphPad Prism 5 was used to plot survival curves and cell survival fraction statistics.

### 4.3. Detection of Cell Proliferation

Ten thousand cells were cultured in a 96-well plate with six replicate wells per group. Once the cells reached the logarithmic growth phase, 10 µL of cell counting kit-8 working solution (CCK-8 kit, BMU106, Abbkine, Wuhan, China) was injected into each well and incubated at 37 degrees Celsius with 5% CO_2_ for 4 h. The experiments were performed in triplicate.

### 4.4. Cell Migration Assay

This study adopted non-extracellular matrix-coated transwell chambers to detect cell migration capacity exclusively, without Matrigel coating to exclude cell invasion behavior. A total of 10,000 cells were cultured in 100 µL of serum-free medium in the upper chamber of the transwell migration assay tube. The lower chamber contained 600 µL of 20% serum medium. After incubating the cells for 48 h at 37 °C with 5% CO_2_, non-migrant cells were removed. The remaining migrating cells were fixed, stained and counted under a microscope (average of 3 fields of view). The migration data were then analyzed.

### 4.5. Cell Scratch Assay

A total of 100,000 cells were cultured well in a six-well plate and allowed to grow to confluence. Once confluent, linear scratches were created in the cell monolayer using a sterile pipette tip (Biosharp, Hefei, Anhui, China). The wound closure process was monitored for 24 h at 37 °C with 5% CO_2_. The width of the wound was measured at 0, 12, and 24 h to assess the migratory capacity of the cells.

### 4.6. Western Blotting

Proteins were extracted from samples using RIPA lysis buffer (WB3100, NCM, Suzhou, China) containing protease (05892970001, Sigma-Aldrich, St. Louis, MA, USA) and phosphatase inhibitors (BL615A, Biosharp, Hefei, China). Equal amounts of protein lysate were separated by electrophoresis on a polyacrylamide gel (BL522A, Biosharp, Hefei, China) and then transferred to a PVDF membrane (Millipore, Burlington, MA, USA). The membranes were blocked and subsequently incubated with primary antibodies against β-actin (4970, Cell Signaling Technology, Danvers, MA, USA), E-cadherin (20874-1-AP, Proteintech, Wuhan, China), N-cadherin (22018-1-AP, Proteintech, Wuhan, China), vimentin (10366-1-AP, Proteintech, Wuhan, China), γ-H2AX (9718, Cell Signaling Technology, MA, USA), and p53 at 4 °C overnight. After washing with TBST (T1081-500, Solarbio, Beijing, China), the membranes were washed and further incubated with HRP-conjugated goat anti-rabbit IgG (H + L) (SA00001-2, Proteintech, Wuhan, China) or HRP-conjugated goat anti-mouse IgG (H + L) (SA00001-1, Proteintech, Wuhan, China) for 1 h at room temperature. Protein bands were visualized using enhanced chemiluminescence (ECL, E422, Vazyme, Nanjing, China) and imaged with a chemiluminescence imaging system (Clinex, Shanghai, China).

### 4.7. Detection of Cell Cycle by Flow Cytometry

Cell suspensions were prepared at 6 h and 24 h after irradiation. Cells were resuspended after centrifugation and fixed in cold 75% ethanol, followed by incubation at 4 degrees Celsius overnight. The fixative was discarded, and the cells were washed with chilled phosphate-buffered saline (PBS). Subsequently, the cells were stained with the Cell Cycle Detection Kit (KeyGen Biotech, KGA512, Nanjing, China) and incubated under dark conditions for 30–60 min at room temperature. Finally, stained cells were filtered through a sieve to remove any debris. A total of 1–2 × 10^6^ cells were collected and detected by a BD FACSCelesta 3-laser 12-color flow cytometer. Red fluorescence at an excitation wavelength of 488 nm was measured, and the results were analyzed by FlowJo v10 software.

### 4.8. Construction of Mouse Tumor Model

All animal experiments were performed in accordance with the ARRIVE 2.0 guidelines and approved by the Institutional Animal Care and Use Committee of Anhui Medical University (protocol code 20252677). For in vivo animal experiments, mice were randomly allocated to experimental groups after grouping by body weight. For the construction of the xenograft mouse model, we prepared a suspension of 25 million A549 cells in 1 mL and injected 200 µL subcutaneously into each nude mouse in the right groin (3 per group). Tumor growth was observed. As the volume reached 60–100 mm^3^, meeting the irradiation requirement, the tumors were irradiated with 6 Gy X-rays. To monitor tumor growth and calculate the tumor volume, tumor size (length, width, height) was measured at an interval of approximately 24 h. In addition, the body weight of mice was also assessed at the same time to monitor weight changes. To mitigate confounding effects arising from inter-animal variation in baseline tumor size, tumor growth data were normalized to the tumor volume measured at the time of irradiation. Pre-defined humane endpoints included tumor volume exceeding 1200 mm^3^, tumor ulceration, or obvious signs of debilitation. Animals meeting any of these criteria were humanely euthanized before the experimental endpoint.

In the tail vein pulmonary colonization model, A549 cells were cultured to the logarithmic growth phase, washed with PBS, trypsinized, and resuspended in PBS. Subsequently, 5 million cells were injected into the tail vein of nude mice (4 per group) using a 0.2 mL injection volume of PBS to induce tumor formation. After 4–8 weeks, when metastasis was suspected, we performed micro-positron emission tomography (PET) imaging using a microPET scanner (Siemens Medical Solutions, Knoxville, TN, USA). Each mouse received 400 μCi of 18F-FDG administered into the tail vein (*n* = 4 per group) and underwent a 5 min PET scan 1 h later. Finally, the mice were sacrificed for the histopathological analysis of metastasis formation through H&E staining (*n* = 4 per group).

### 4.9. H&E Staining

Formalin-fixed and paraffin-embedded lung tissue specimens were serially sectioned at a thickness of 5 μm for routine hematoxylin and eosin (H&E) staining. Briefly, sections were deparaffinized in xylene I and xylene II for 26 min each, rehydrated through a graded ethanol series (100%, 95%, 85%, and 75%) for 6 min at each concentration, and thoroughly rinsed with running tap water. The sections were stained with hematoxylin for 7 min, differentiated in acidic alcohol for 2 s, and treated with bluing solution for 1 min, followed by thorough rinsing. After the complete removal of residual water, sections were counterstained with eosin for 2 min. Subsequently, the sections were dehydrated three times with absolute ethanol (1 min per time), cleared with n-butanol for 2 min, and further cleared in xylene I and xylene II for 5 min each. Finally, all sections were mounted with neutral resin. Whole-slide images were acquired using a digital pathology scanner (AF-KL-20-8, KFBIO, Changsha, China) under bright-field illumination for subsequent histological observation and analysis.

### 4.10. Statistical Analysis

All experiments were performed with at least three independent biological replicates unless otherwise stated. Data are presented as the mean ± standard deviation (SD). Technical replicates were defined as parallel wells or culture dishes within a single independent experiment, and their values were averaged before statistical analysis. Independent biological replicates were defined as separate experiments performed on different days using cells at different passage numbers. The experimental unit was defined as one independent cell culture experiment for in vitro assays and one individual mouse for in vivo studies. The number of biological replicates is indicated in each figure legend (e.g., *n* = 3 independent biological replicates, each with three technical replicates).

All quantitative measurements, including colony counting, migration assays, and the histopathological assessment of H&E-stained lung sections, were performed in a blinded manner. Investigators and pathologists were blinded to group allocation during evaluation.

Statistical analyses were performed using GraphPad Prism software. A two-tailed unpaired Student’s *t*-test was used for two-group comparisons. For comparisons among multiple groups, a one-way ANOVA followed by Tukey’s post hoc test was used. For experiments involving two factors, a two-way ANOVA followed by Tukey’s post hoc test was used. Multiple comparisons were corrected using Tukey’s test. *p* < 0.05 was considered statistically significant. * *p* < 0.05, ** *p* < 0.01, *** *p* < 0.001.

## 5. Conclusions

Collectively, this study investigated the long-term oncogenic effects of low-dose radiation on pre-existing lung adenocarcinoma cells. Our results demonstrate that low-dose irradiation induces persistent DNA damage, cell cycle dysfunction, glycolytic reprogramming, and epithelial–mesenchymal transition, thereby enhancing the pulmonary colonization capacity of long-term passaged A549 cells.

Distinct from previous studies that primarily focus on radiation-induced de novo carcinogenesis in normal tissues, the present work provides compelling preclinical evidence that clinically relevant cumulative low-dose radiation predominantly promotes the progression of existing tumor lesions, rather than merely initiating new tumor formation.

The present findings are exploratory and hypothesis-generating in nature and remain limited to the A549 cell model. Direct extrapolation to low-dose CT exposure is not warranted and requires further validation in additional models.

## Data Availability

The datasets generated or analyzed during the study are available from the corresponding author on reasonable request.

## Abbreviations

The following abbreviations are used in this manuscript:

LDCT	Low-Dose Computed Tomography
EMT	Epithelial–Mesenchymal Transition
DSB	Double-Strand Break
PVDF	Polyvinylidene Difluoride
HE	Hematoxylin and Eosin
ECL	Enhanced Chemiluminescence
PBS	Phosphate-Buffered Saline
PET	Positron Emission Tomography
γ-H2AX	Phosphorylated Histone H2AX
PKM2	Pyruvate Kinase M2
18F-FDG	^18^F-Fluorodeoxyglucose
GLUT-1	Glucose Transporter 1
HK-II	Hexokinase II
ATM	Ataxia–Telangiectasia Mutated
ATR	Ataxia–Telangiectasia and Rad3-Related
CHK1/2	Checkpoint Kinase 1/2
STR	Short Tandem Repeat
DMEM	Dulbecco’s Modified Eagle Medium
FBS	Fetal Bovine Serum
RIPA	Radio-Immunoprecipitation Assay
